# Combination PPAR*γ* and RXR Agonist Treatment in Melanoma Cells: Functional Importance of S100A2

**DOI:** 10.1155/2010/729876

**Published:** 2009-10-18

**Authors:** Joshua P. Klopper, Vibha Sharma, Reid Bissonnette, Bryan R. Haugen

**Affiliations:** ^1^Division of Endocrinology, Metabolism and Diabetes, Department of Medicine, University of Colorado Denver, Aurora, CO 80045, USA; ^2^University of Colorado Cancer Center, University of Colorado Denver, Aurora, CO 80045, USA; ^3^Department of Molecular Oncology, Ligand Pharmaceuticals, San Diego, CA 92121, USA

## Abstract

Nuclear hormone receptors, including RXR and PPAR*γ*, represent novel therapeutic targets in melanoma. We have previously shown that the DRO subline of the amelanotic melanoma A375 responds to rexinoid and thiazolidinedione (TZD) treatment *in vitro* and *in vivo*. We performed microarray analysis of A375(DRO) after TZD and combination rexinoid/TZD treatment in which the calcium binding protein S100A2 had increased expression after rexinoid or TZD treatment and a synergistic increase to combination treatment. Increased S100A2 expression is dependent on an intact PPAR*γ* receptor, but it is not sufficient to mediate the antiproliferative effects of rexinoid/TZD treatment. Over expression of S100A2 enhanced the effect of rexinoid and TZD treatment while inhibition of S100A2 expression attenuated the response to rexinoid/TZD treatment, suggesting that S100A2 is necessary for optimal response to RXR and PPAR*γ* activation by respective ligands. In summary, we have identified potential downstream mediators of rexinoid and TZD treatment in a poorly differentiated melanoma and found that alterations in S100A2 expression affect RXR and PPAR*γ* signaling in A375(DRO) cells. These studies provide insight into potential mechanisms of tumor response or resistance to these novel therapies.

## 1. Introduction

Melanoma represents a significant public health problem with a rising incidence over the last 3 decades [1]. More than 7700 patients will die of this disease annually, almost all with metastases [2]. The median survival in patients with metastatic disease is 7–9 months [3]. While some prognostic factors correlate with a more favorable prognosis, such as lack of visceral metastases, younger age, and treatment with biochemotherapy, the 5–10-year survival rates still remain less than 20% [4]. Thus, a search for novel therapies is warranted in this aggressive disease given the suboptimal choices available. 

We have reported the efficacy of rexinoid, thiazolidinedione, and combination therapy in the melanoma cell line A375(DRO) (DRO was originally thought to be an anaplastic thyroid cancer cell line) [5–7]. Additionally, we have shown that RXR and PPAR*γ* receptors are necessary for optimal response to rexinoid or TZD therapies, as knock down of either receptor attenuates the antiproliferative response to its own ligand alone or the ligand of its heterodimer partner [8]. 

In this report, we explore potential downstream mediators of the rexinoid and TZD treatment effect in the A375(DRO) melanoma cancer cell line using comparative gene expression microarray analysis. We have identified the calcium binding protein S100A2 as a potential mediator of rexinoid and TZD signaling in melanoma. S100A2 is one of 24 members of S100 proteins that regulate cellular processes including neoplasia and has significantly increased gene expression in A375(DRO) with rexinoid and TZD, while a synergistic effect is seen with combination therapy [9].

## 2. Materials and Methods

### 2.1. Cell Line and Chemicals

A375(DRO) was provided by Dr. G.J. Juillard (University of California at Los Angeles, Los Angeles, CA). DRO was previously thought to be derived from an anaplastic thyroid cancer. We have shown that it is genetically identical to the melanoma cell line A375 and is therefore designated as a subline of A375, A375(DRO) [7, 8]. A375(DRO) was grown in RPMI 1640 (Invitrogen Corporation) supplemented with 2% fetal bovine serum (Hyclone) and 0.5% penicillin/streptomycin. LGD1069 was provided by Ligand Pharmaceuticals (San Diego, CA), and Rosiglitazone (ROSI) was provided by GlaxoSmithKline.

### 2.2. Microarray Analysis

Four million A375(DRO) cells were plated in triplicate into 100 mm plates and incubated overnight. The next day, the medium was changed, and medium with volume equivalent vehicle (DMSO) or 1 *μ*mol/L of LGD1069, ROSI, or the combination (500 nM of each) was added in the set of cells to incubate for 24 hours. RNA was extracted from treated cells using the QIAGEN RNeasy Mini Kit and was quantified by standard spectrophotometry. RNA integrity was verified by gel electrophoresis using an Agilent 2100 Bioanalyzer. Total RNA (5 *μ*g) was converted to ds-cDNA using the Superscript Choice System (Life Technologies). A high-pressure liquid chromatography-purified T7-(dT)_24_ primer was used to initiate the cDNA reverse transcription. After the synthesis of both strands of DNA, the ds-cDNA was extracted with phenol-chloroform-isoamyl alcohol and recovered by ethanol precipitation. Next, in vitro transcription of cRNA was done and the transcript underwent biotin labeling using an RNA Transcript Labeling Kit (Enzo). Biotin-labeled cRNA was purified using the QIAGEN RNeasy Mini Kit. The RNA was then fragmented by incubating the cRNA at 94°C for 35 minutes to allow optimal hybridization to the cDNA oligonucleotide array. We used the Affymetrix GeneChip Human Genome U133A platform, and all gene chip processing and analyses occurred in the UCHSC Affymetrix microarray core facility. Each condition was run in triplicate from three independent experiments. Data analysis, including background adjustment and normalization, was done using Affymetrix GeneSpring software. For microarray analysis, those genes that had at least a >2-fold change in more than three of six gene chips between ligand and vehicle treatment of DRO cells were selected as significant. Significance was determined by a one-way ANOVA (*P* < .05).

### 2.3. Quantitative Reverse Transcription-PCR (qRT-PCR)

Total RNA was isolated from A375(DRO) in single samples under the same conditions used for the microarray experiment using the RNeasy Mini Kit (Quiagen, Valencia, CA) as per the manufacturer's protocol. The mRNA for S100A2 was measured by real-time Quantitative RT-PCR using ABI PRISM 7700. The sequences of forward and reverse primers as designed by Primer Express (PE ABI) were 5′-TTCCTGGGTCTGTCTCTGCC-3′ and 5′-AGCGCCTGCTCCAGAGAAC-3′. 

 The TaqMan fluorogenic probe used was 6FAM-TGGTCTGCCACAGATCCATGATGTGC-TAMRA. 

Amplification reactions, thermal cycling conditions, and generation of a standard curve have been described previously [6].

### 2.4. S100A2 Overexpression

Human S100A2 in pcDNA3 vector was the generous gift from Professor C. Heizmann (University of Zurich). A375(DRO) cells were stably transfected with S100A2 in pcDNA3 vector and empty vector using lipofectamine method in 6-well cell culture plates (4 *μ*g/well) as previously described [10]. Thereafter stable clones were selected and continuously cultured in 150 *μ*g/mL G418(Gibco/BRL).

### 2.5. shRNA

We used a lentiviral mediated shRNA system from Sigma (St. Louis, MO) and followed the manufacturer's protocol. Lentiviral particles contain shRNA toward S100A2 or PPAR*γ* or RXR*γ*-specific sequences as well as a scrambled (SCR) sequence that consists of 5 nucleotides that do not match any known gene transcript in both the murine and human genome. The infected cells are selected by a puromycin resistance and then assessed for correct insertion/RNA inhibition by qRT-PCR or western blot for S100A2, PPAR*γ*, or RXR*γ*. The concentration of puromycin used to select for DNA construct incorporation cells was 0.4 *μ*g/mL.

### 2.6. Western Blot Analysis

Whole cell protein extracts were obtained from A375(DRO) under conditions of volume equivalent vehicle, LGD1069/ROSI combination treatment, and with overexpressed S100A2 or shRNA directed at S100A2. The protein content of lysates was measured using a commercial protein assay kit (DC from Bio-Rad). Diluted samples containing equal amounts of protein (60 *μ*g) were mixed with 2x Laemmli sample buffer (Bio-Rad Laboratories). Proteins were separated on a 10% SDS-polyacrylamide gel and transferred to polyvinylidene difluoride membranes. The membranes were blocked with 1x TBST (20 mmol/L Tris-HCl (pH 7.6), 8.5% NaCl, and 0.1% Tween 20) containing 5% nonfat dry milk at room temperature for 2 hours and incubated in the appropriate primary antibody in 1x TBST containing 5% nonfat dry milk at 4°C overnight. S100A2 protein antibodies (Sigma S6797), RXR*α* (sc 553 and D-20), RXR*β* (sc 831 and C-20), and RXR*γ* (sc Y-20 and JC-555) receptor antibodies were used at a concentration of 1 : 1000, and PPAR*γ* (sc 7196 and H-100) rabbit polyclonal antibody was used at 1 : 500. After washing, membranes were incubated for 1 hour at room temperature with antirabbit IgG conjugated to horseradish peroxidase at a 1 : 5000 dilution for RXRs and 1 : 1000 for PPAR*γ* (GE Healthcare UK). *β*-Actin was probed for loading control. The enhanced chemiluminescence detection reagent from Amersham Biosciences was used for immunodetection.

### 2.7. Cell Growth and Proliferation

A375(DRO) cells at baseline, with S100A2 overexpressed, with infected SCR shRNA, and with shS100A2 cells were grown to approximately 80% confluence in 100 mm tissue culture plates. Cells were then harvested using Trypsin-EDTA (Invitrogen Corporation, Carlsbad, CA) and counted using a hemocytometer. Cells were then transferred to a 96-well plate at a concentration of 500 cells/200 *μ*L of media. Each row of eight wells received the same cell type and subsequently the same drug. After cells were plated, media with the appropriate concentration of ligand or equivalent volume of vehicle was added to each well. Cells were treated with volume equivalent vehicle, 1 *μ*M LGD1069, 1 *μ*M rosiglitazone, or the 1 *μ*M combination (500 nM of each). Fresh media with vehicle or ligand was added every 72 hours. At the completion of 6 days, cell proliferation was assessed following the manufacturers instructions using the CellTiter 96 Aqueous Non-Radioactive Cell Proliferation Assay (Promega, Madison, WI). Following a two-hour incubation at 37°C, each plate was analyzed by a MRX Micro plate Reader (Dynatech Laboratories, Chantilly, VA) using Revelation software.

### 2.8. Statistics

Cell growth between control and treatment conditions quantified using the group mean ± SE and significance was compared between control and treatment conditions with a Student's *t*-test between conditions (SISA online statistical tool).

## 3. Results

### 3.1. Microarray Analysis of LGD1069/ROSI-Treated A375(DRO) Cells

The antiproliferative effects of rexinoid and TZD treatment on A375(DRO) occur at or beyond six days of treatment [5]. However, we chose to analyze gene expression changes in A375(DRO) at 24 hours since RXR*γ* and PPAR*γ* are nuclear hormone receptors, and we would predict direct gene expression effects of these liganded transcription factors to occur early. The results of the LGD1069 1 *μ*M treatment arm have been previously published [6]. Microarray analysis revealed that the combination rexinoid/TZD treatment resulted in 212 genes with increased expression and 1050 genes with decreased expression (one-way ANOVA *P* < .05). These genes broadly fell into the categories of cell growth, nucleic acid binding, and cell signal transduction. The 20 genes with the largest change in expression after treatment are listed in Tables 1, 2, 3, and 4 (excluding affymetrix specific cDNA sequences without related searchable genes). The complete data set is in the supplementary materials (see Supplementary Material available online at doi:10.1155/2010/729876). Four genes were upregulated by rexinoid/TZD combination therapy greater than 20-fold: TIE1 (121.5-fold), S100A2 (69.1-fold), ILB-1 (40.1-fold), and ANGPTL4 (32.2-fold) (Table 5). Of these genes, S100A2 was increased by both the rexinoid (3.4-fold) and TZD (4.9-fold) but also demonstrated a synergistic stimulation (69.1-fold) with the combination treatment. In addition, 171 genes had increased expression and 1006 genes had decreased expression by at least 2-fold with ROSI alone (Tables 1, 2, 3, and 4—one-way ANOVA *P* < .05). Based upon the significant increase of S100A2 mRNA levels with each ligand alone and the synergistic increase with combination therapy, we performed additional experiments with S100A2 to define its role in mediating the effects of combination rexinoid/TZD treatment in melanoma cells. We have previously published confirmation of ANGPTL4 regulation by rexinoids [6]. The effects of TZD and rexinoids on TIE-1 and ILB-1 mRNA and protein expression have not yet been confirmed by other methods.

### 3.2. An Intact RXR*γ* and PPAR*γ* Receptor Is Required for Optimal S100A2 Expression

**Figure 1:**
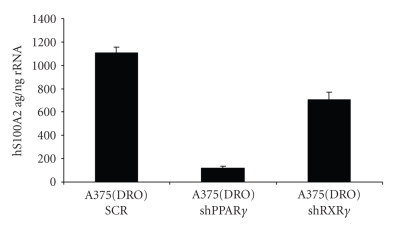
S100A2 mRNA stimulation by TZD/rexinoid treatment is dependent on intact PPAR*γ* and RXR*γ*. One microgram of total RNA was used for the S100A2 quantitative reverse transcription-PCR analysis (ABIPRISM7700; Perkin-Elmer), and absolute values were derived from a standard curve using a known amount of sense strand RNA (ag, attograms of sense strand RNA). Isoform RNA was normalized to total input RNA (18s rRNA measured from 1 ng of total RNA). A375(DRO) cells were infected with either shPPAR*γ* or shRXR*γ* lenteviral particles and then treated with LGD1069/ROSI 1 *μ*M for 24 hours. S100A2 mRNA levels were compared to levels from A375(DRO) cells infected with the shSCR control under the same treatment conditions.

S100A2 levels were measured after treatment of A375(DRO) with 1 *μ*M combination of LGD1069 and ROSI (500 nM each). To determine the relative contribution of PPAR*γ* and RXR*γ*, we compared control cells stably infected with scrambled shRNA with sublines stably infected with shRNA against PPAR*γ* and RXR*γ* which greatly reduced the levels of each receptor [8]. Figure 1 shows that the rexinoid/TZD-induced expression of S100A2 was attenuated by lack of either receptor. PPAR*γ* appears to have the greatest effect on this response.

### 3.3. S100A2 Overexpression Enhances the Antiproliferative Response to LGD1069 and ROSI Treatment

**Figure 2:**
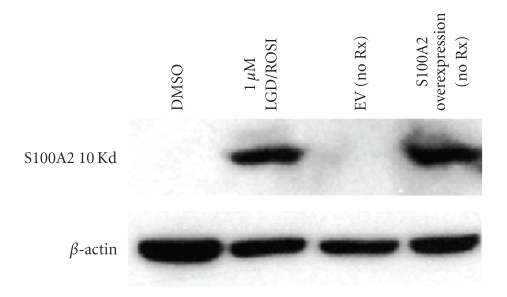
Protein expression of S100A2 in A375(DRO) cells. 60 *μ*g of nuclear protein extract from A375(DRO) before (DMSO—lane 1) and after combination treatment (lane 2) or transfected with empty vector (EV—lane 3) or S100A2 in pcDNA3 vector with no treatment (lane 4) was size-separated on a 10% SDS-PAGE gel and transferred to nitrocellulose. The blot was blocked with 10% nonfat milk and incubated with S100A2 receptor antibodies (sc Y-20). *β*-Actin was measured as a loading control.

**Figure 3:**
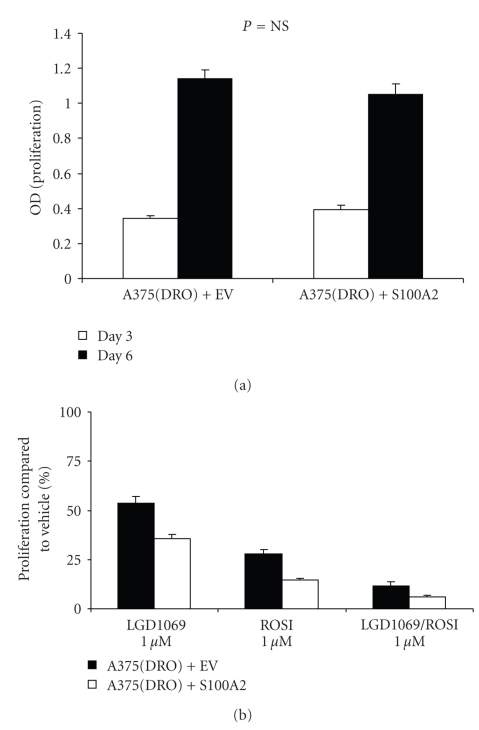
Proliferation of A375(DRO) cells overexpressing S100A2. Cells were grown in 2% fetal bovine serum RPMI in the presence of 1 *μ*mol/L of LGD1069, TZD, or the combination for 6 days. Cell growth was analyzed using a nonradioactive cell proliferation assay. Proliferation was compared directly between EV and S100A2 (a) and then to that of cells grown in volume equivalent vehicle (set at 100% which represents cell growth in control conditions) (b). Proliferation was significantly decreased in cells with S100A2 overexpression compared to EV for all treatment conditions compared to control. Columns mean; bars, SEM.

S100A2 protein was overexpressed in A375(DRO) cells, and the empty vector (EV) was used as a control. Figure 2 shows that the levels of S100A2 protein in the overexpressing subline (S100A2) are similar to levels seen after treating A375(DRO) cells with rexinoid/TZD combination. After plating equivalent numbers of control (A375(DRO) + EV) and S100A2 overexpressing cells, we observed no difference in growth rate at 3 and 6 days (Figure 3(a)). However, with 6 days of 1 *μ*M LGD1069, 1 *μ*M ROSI, or 1 *μ*M combination therapy the S100A2 overexpressing cells had a significant decrease in proliferation compared to the EV cells relative to vehicle treatment (64% versus 46% for LGD1069, 86% versus 72% for ROSI, and 94% versus 88% for the combination; *P* ≤ .008 for all conditions; Figure 3(b)).

### 3.4. Knock Down of S100A2 Attenuates the Growth Inhibition of A375(DRO) Cells by Rexinoid, TZD, and Combination Treatment

**Figure 4:**
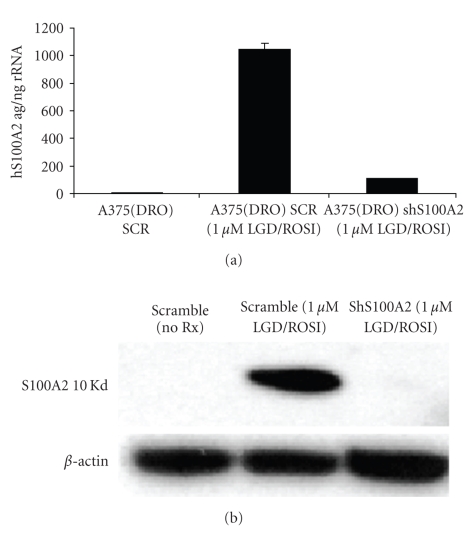
shS100A2 decreases S100A2 mRNA and protein in A375(DRO) cells. qRT-PCR and Western blot of S100A2. (a) One microgram of total RNA was used for the S100A2 quantitative reverse transcription-PCR analysis (ABIPRISM7700; Perkin-Elmer), and absolute values were derived from a standard curve using a known amount of sense strand RNA (ag, attograms of sense strand RNA). Isoform RNA was normalized to total input RNA (18s rRNA measured from 1 ng of total RNA). (b) 60 *μ*g of nuclear protein extract from A375(DRO), the SCR shRNA infected control cell, and shS100A2 infected cells. Proteins were size-separated on a 10% SDS-PAGE gel and transferred to nitrocellulose. The blot was blocked with 10% nonfat milk and incubated with S100A2 primary antibody (sc Y20) and then secondary antibody with antimouse IgG conjugated to horse-radish peroxidase as previously described. *β*-actin was measured as a loading control.

**Figure 5:**
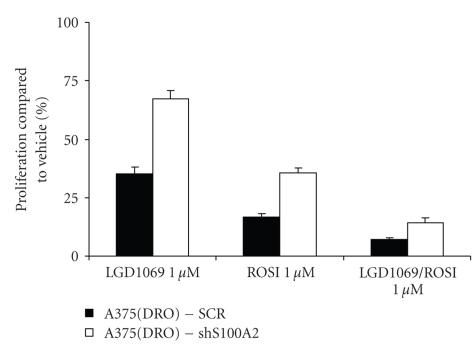
Knock down of S100A2 blunts the antiproliferative effect of TZD and rexinoids in A375(DR) cells. A375(DRO), the SCR infected, and an shS100A2 infected subline were grown in 2% fetal bovine serum RPMI in the presence of 1 *μ*mol/L of LGD1069, TZD, or the combination for 6 days. Cell growth was analyzed using a nonradioactive cell proliferation assay. Proliferation was compared to that of cells grown in volume equivalent vehicle (DMSO—set at 100%). Proliferation was statistically significantly attenuated compared to the A375(DRO) SCR subline in all treatment conditions. Columns, mean; bars, SEM.

**Figure 6:**
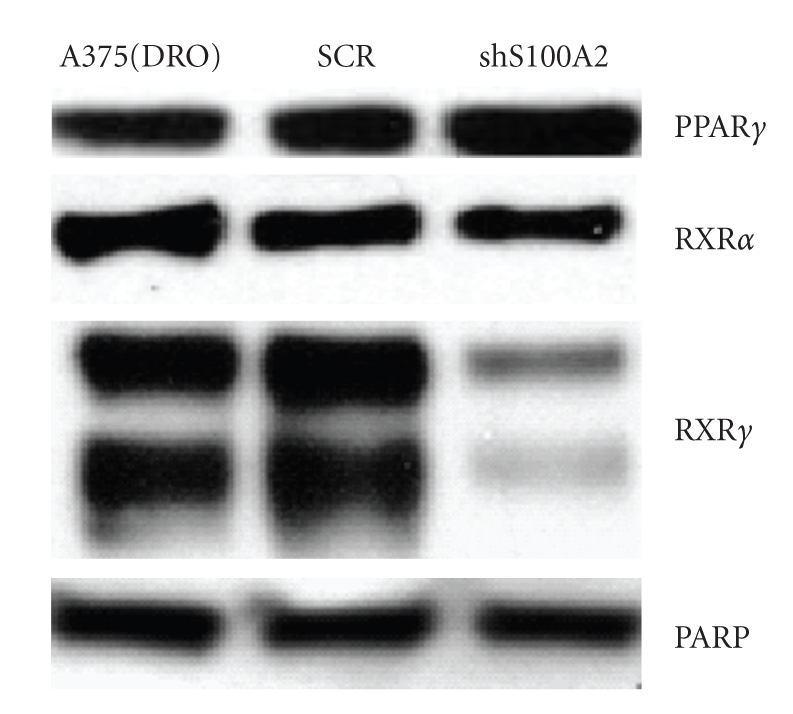
Western blot of rexinoid receptors in A375(DRO) cells after knock down of S100A2. 60 *μ*g of nuclear protein extract from A375(DRO), the SCR shRNA-infected control cell, and a clone of shS100A2 infected cells were size-separated on a 10% SDS-PAGE gel and transferred to nitrocellulose. The blot was blocked with 10% nonfat milk and incubated with PPAR*γ*, RXR*γ*, and RXR*α* primary antibodies and then secondary antibody with antirabbit IgG conjugated to horse-radish peroxidase as previously described. PARP was measured as a loading control.

Knock down of S100A2 with specific shRNA did not affect growth of A375(DRO) cells (data not shown). S100A2 shRNA stably expressed in A375(DRO) cells resulted in a decrease in S100A2 mRNA and protein expression after treatment with LGD1069/ROSI (Figure 4). Stable expression of scrambled shRNA (SCR) had no effect. Figure 5 shows that melanoma cells stably transfected with S100A2 shRNA had significantly blunted growth suppression by treatment with rexinoid, TZD, or combination (*P* < .002 for all shS100A2 versus SCR treatment conditions). These data indicate that S100A2 is required to mediate the growth suppressive effects of TZD and rexinoids in melanoma cells. Western blot analysis of the cells expressing S100A2 shRNA revealed decreased levels of RXR*γ* but RXR*α* and PPAR*γ* protein levels were unaffected (Figure 6). RXR*β* protein levels are very low in these cells [11]. Analysis of shS100A2 and RXR*γ* sequences revealed no sequence homology.

## 4. Discussion

In this report, we have examined global gene expression in a poorly differentiated cancer model, the amelanotic melanoma cell line A375(DRO), after treatment with PPAR*γ* and RXR ligands. S100A2 was shown to be a potentially important target based on increased levels with rexinoid or TZD treatment and synergistically increased levels with combination therapy. Furthermore, S100A2 appears to be required for the maximal antiproliferative effects of rexinoids and TZD in these melanoma cells. 

The S100 proteins have a broad range of intracellular functions including the regulation of protein phosphorylation and enzyme activity, calcium homeostasis, regulation of cytoskeletal proteins, and transcriptional factors [12]. S100 proteins appear to regulate tumorigenesis. For example, S100A2 proteins enhance p53 transcriptional activity whereas S100A4 increases p53 apoptosis in models of adenocarcinoma, osteosarcoma, and oral carcinoma [12–14]. Thus, a relative imbalance of S100 proteins may promote or inhibit neoplastic transformation or progression. S100A2 seems to have a variable pattern of expression with some evidence pointing to higher expression in normal tissues and early or premalignant issues, but other types of cancer, such as lung cancer, have a higher expression in advanced lesions that correlate with poorer clinical prognosis. However, even within the lung cancer literature, there is disagreement regarding the prognostic significance of S100A2 expression [13, 15, 16]. 

In melanoma, S100 proteins may play a critical role in regulating the transformation of nevi to melanoma. S100A4 levels are lower in metastatic melanoma compared with primary tumors, while S100A7, S100A8, and S100A9 levels appear to be higher in malignant melanoma compared with normal melanocytes [17]. In a study of 105 patients with stage IV melanoma, elevated serum levels of S100B were associated with a significantly shorter survival [18].

 S100A2 expression is higher in premalignant nevi than in cells from primary melanoma tumors or metastases suggesting that loss of S100A2 may be important for neoplastic transformation [19]. In an in vitro model of uveal melanoma, S100A2 gene expression was significantly up-regulated by the methyltransferase inhibitor decitabine, which was correlated with cell death [20]. We have demonstrated that S100A2 levels are low in the A375(DRO) melanoma cell line, and these levels are increased by treatment with PPAR*γ* and RXR agonists, which is associated with a significant reduction in growth and increase in apoptosis [8]. 

Though other S100 proteins have been shown to increase with retinoid therapy in models of cancer including teratocarcinoma, breast cancer, and gastric carcinoma [21–23], this is the first report of increased S100A2 expression by either rexinoids or TZDs. We have previously demonstrated that, in A375(DRO), the combination of LGD1069 and ROSI synergistically decreases in vitro cell proliferation and *in vivo* tumor growth [5, 8]. Our data indicates that S100A2 is necessary to mediate the antiproliferative effects of rexinoid and TZD treatment but is not sufficient to mediate this effect. However, with overexpression of S100A2, we observe an enhanced effect of rexinoid and TZD treatment on the melanoma cells. 

This observed relative resistance to rexinoid and TZD treatment with shS100A2 was found in conjunction with decreased RXR*γ* protein levels (though the shS100A2 sequence does not overlap with RXR*γ*). We have previously shown that decreasing RXR*γ* by shRXR*γ* in A375(DRO) decreases response to rexinoid, TZD, and the combination [8]. Modulators of retinoid receptors have been described in melanoma and include HSP 90 and Cyclophilin B [24], but we were unable to find any direct link between S100A2 expression and RXR regulation. It appears as if the presence of S100A2 is important for optimal RXR*γ* expression, but this is most likely not a direct interaction as measurable and at least partially functional RXR*γ* (as evidenced by response to LGD1069) was seen after shS100A2 infection. Further studies will be needed to elucidate the exact interaction of S100A2 and retinoid receptors. 

In summary, we have performed a microarray analysis of a poorly differentiated melanoma after rexinoid and TZD treatment. S100A2 gene expression is significantly increased by both rexinoid and TZD treatment alone and is synergistically increased by combination therapy. S100A2 is necessary for the maximal antiproliferative effect of rexinoid and TZD in this model, but it is not sufficient to mediate this effect. 

## Supplementary Material

Complete microarray data set of A375(DRO) after rexinoid, TZD and combination therapy for 24 hours using the Affymetrix GeneChip Human Genome U133A platform.

## Figures and Tables

**Table 1:** Microarray Analysis of A375(DRO) cells after TZD/rexinoid combination treatment—2-fold up regulated genes. A375(DRO) cells were treated in vitro with 1 *μ*mol/L of PIO or combination LGD1069/PIO for 24 hours compared with volume-equivalent vehicle in triplicate with a cutoff of a 2-fold change (*P* < .5, one-way ANOVA). Gene symbols were derived from the Affymetrix web site.

Gene Symbol	fold change	GenBank ID
TIE1	121.5	NM_005424
S100A2	69.1	NM_005978
IL1B	40.1	NM_000576
ANGPTL4	32.2	NM_001039667
AREG	18.2	NM_001657
RPL37A	12.6	NM_000998
CORO2B	10.9	NM_006091
RPL27A	8.7	NM_000990
INHBA	7.6	NM_002192
ST3GAL1	5.4	NM_003033
TXN	5.0	NM_003329
ITGA3	5.0	NM_002204
FGFR2	5.0	NM_000141
GEM	4.9	NM_005261
COL5A2	4.9	NM_000393
RPL38	4.8	NM_000999
PMEPA1	4.7	NM_020182
MCL1	4.5	NM_021960
RPL38	4.2	NM_000999
SMARCA2	3.8	NM_003070

**Table 2:** Microarray Analysis of A375(DRO) cells after TZD/rexinoid combination treatment—2-fold down regulated genes.

Gene Symbol	fold change	GenBank ID
ABAT	−17.5	NM_000663
TSFM	−10.4	NM_005726
XRCC4	−9.0	NM_003401
NUDT1	−8.9	NM_002452
ZIC1	−8.3	NM_003412
BARD1	−8.2	NM_000465
CDC25A	−8.2	NM_001789
MAP9	−8.1	NM_001039580
QPCT	−7.9	NM_012413
C12orf52	−7.8	NM_032848
PTCD2	−7.3	NM_024754
TLE1	−6.8	NM_005077
MYO6	−6.4	NM_004999
WDR59	−6.0	NM_030581
RARS	−5.6	NM_002887
BIN1	−5.2	NM_004305
WBP1	−5.2	NM_012477
CSGALNACT1	−5.1	NM_018371
CAMTA1	−5.1	NM_015215
TRIM23	−5.1	NM_001656

**Table 3:** Microarray Analysis of A375(DRO) cells after TZD/rexinoid combination treatment—2-fold up regulated with ROSI alone.

Gene Symbol	fold change	GenBank ID
ANGPTL4	29.4	NM_001039667
TIE1	13.1	NM_005424
RPL27A	10.9	NM_000990
IL1B	9.3	NM_000576
RPL37A	8.4	NM_000998
AREG	6.1	NM_001657
RPL38	6.1	NM_000999
TACR1	5.6	NM_001058
TXN	5.5	NM_003329
MCL1	5.5	NM_021960
PGK1	5.5	NM_000291
RPL38	5.4	NM_000999
FGFR2	5.2	NM_000141
LOC653505	5.0	NM_001123068
RAB2A	5.0	NM_002865
S100A2	4.9	NM_005978
LOC100131637	4.8	XM_001716033
GBA3	4.7	NM_020973
JUN	4.4	NM_002228
SMARCA2	4.2	NM_003070

**Table 4:** Microarray Analysis of A375(DRO) cells after TZD/rexinoid combination treatment—2-fold down regulated with ROSI alone.

Gene Symbol	fold change	GenBank ID
ORC4L	−15.9	NM_002552
TAF12	−8.9	NM_005644
ZNF443	−8.1	NM_005815
HIGD1A	−8.0	NM_032775
CYB5R2	−7.9	NM_001039580
DAZAP2	−7.5	NM_005726
STK32B	−7.1	NM_024754
ZBTB43	−6.7	NM_014007
CAMTA1	−6.6	NM_015215
HCG4	−6.4	NR_002139
BSCL2 /// HNRNPUL2	−6.3	NM_001079559
SLC39A4	−6.0	NM_017414
DZIP3	−6.0	NM_014648
MSRB2	−6.0	NM_018009
KLHL22	−5.8	NM_032848
MPP5	−5.7	NM_024899
RNF41	−5.6	NM_005785
VRK3	−5.5	NM_030581
ABAT	−5.4	NM_000663
BRCA2	−5.3	NM_000059
RARS	−5.3	NM_002887

**Table 5:** Four genes with the highest mRNA stimulation after combination TZD/rexinoid treatment.

	TIE 1	S100A2	IL1B	ANGPTL4
LGD1069/ROSI	121.5	69.1	40.1	32.2
ROSI	13.1	4.9	9.3	29.4
LGD1069*	<2	3.4	<2	6.5

*data published in [6].
